# Comparison of light transmission aggregometry in patients with bleeding disorder of unknown cause and healthy blood donors

**DOI:** 10.1016/j.rpth.2025.103214

**Published:** 2025-10-10

**Authors:** Lena-Theresa Fanenbruck, Michael Metze, Maria Weise, Martin Federbusch, Roland Siegemund, Martin Reinhard Henschler, Annelie Siegemund, Sirak Petros, Christian Pfrepper

**Affiliations:** 1 Division of Hemostaseology, University of Leipzig Medical Center, Leipzig, Germany; 2 Department of Cardiology, University of Leipzig Medical Center, Leipzig, Germany; 3 Institute of Laboratory Medicine, University of Leipzig Medical Center, Leipzig, Germany; 4 Medical Intensive Care Unit, University of Leipzig Medical Center, Leipzig, Germany; 5 Institute of Transfusion Medicine, University of Leipzig Medical Center, Leipzig, Germany

**Keywords:** ADP, arachidonic acid, bleeding of unknown cause, blood donor, light transmission aggregometry, platelet function test

## Abstract

**Background:**

Light transmission aggregometry (LTA) plays an important role in the detection of platelet function disorders.

**Objectives:**

This study compared different agonist concentrations to detect patients with bleeding disorder of unknown cause (BDUC) with healthy blood donors.

**Methods:**

We retrospectively evaluated LTA performed in patients with BDUC and compared the results with prospectively collected LTA measurements from healthy blood donors. LTA was assessed with 2 μM, 5 μM, and 20 μM adenine diphosphate (ADP) in both groups; 1.0 mM arachidonic acid (AA) and 5 μM epinephrine in persons with BDUC; 1.0 mM and 1.5 mM AA and 5 μM and 10μM epinephrine in healthy blood donors.

**Results:**

After induction with 2 μM ADP, 43.8% of persons with BDUC and 24.3% of the healthy blood donors had abnormal maximum aggregation, but only 5.8% and 4.6% after induction with 5 μM ADP, respectively. Persons with BDUC had a higher proportion of abnormal maximum aggregation after induction with 1 mM AA (18.5%) compared to healthy donors (3.6%). No difference was observed after epinephrine induction. The sensitivity of an abnormal International Society on Thrombosis and Haemostasis Bleeding Assessment Tool score to predict an abnormal LTA was <22% and the negative predictive value >70%.

**Conclusion:**

This study reveals a high proportion of abnormal LTA results in persons with BDUC and healthy blood donors after induction with low concentrations of ADP and AA. The recommendations for those concentrations in guidelines for platelet function testing should be reassessed.

## Essentials

• The optimal concentration of agonists for light transmission aggregometry (LTA) is controversial.
• LTA of patients with bleeding disorder of unknown cause (BDUC) was compared with that of blood donors.
• A high proportion of people with BDUC and blood donors had abnormal LTA with 2 μM adenosine diphosphate and 1 mM arachidonic acid.
• The recommendations for low agonist concentrations in guidelines for LTA should be reassessed.

## 1 Introduction

Mild to moderate bleeding disorders encompass conditions with mild mucocutaneous bleeding and moderate hemorrhage after hemostatic challenges [1,2]. In up to 60 % of cases, no underlying cause is identified despite thorough laboratory evaluation; these patients are classified as having a bleeding disorder of unknown cause (BDUC). The clinical phenotype of BDUC resembles that of platelet function defects and mild coagulation factor deficiencies [1,2]. Platelet function disorders (PFDs), caused by abnormalities in platelet receptors, signaling pathways, or storage granules, typically present with mucocutaneous bleeding, surgical bleeding, or postpartum hemorrhage and require specialized laboratory diagnostics for confirmation [1,3].

In the diagnostic evaluation of PFDs, clinicians frequently face significant challenges related to limited standardization [4], technical variability [5], and the heterogeneity of the patient population [6]. Since its development in the 1960s, light transmission aggregometry (LTA) has been considered the gold standard for the diagnosis of congenital and acquired PFDs [7,8]. This *in vitro* test measures the ability of platelets to aggregate in response to various agonists such as adenosine diphosphate (ADP), epinephrine (EPI), and arachidonic acid (AA) [9,10] by analyzing changes in light transmission of platelet-rich plasma. By adding different agonist concentrations, LTA can evaluate the aggregation behavior of platelets based on various parameters, such as aggregation rate, lag phase, shape change, and presence of disaggregation. However, despite its widespread use, LTA is hindered by several limitations [8], including poor standardization, inconsistencies in international laboratory practice [4,11], and difficulties in interpretation of platelet aggregation curves [12].

To assess platelet function in patients with suspected PFD, current guidelines of the International Society on Thrombosis and Hemostasis (ISTH) and Society of Thrombosis and Haemostasis Research (GTH) [13] recommend the use of various agonists at different concentrations [14,15]. Despite the continued lack of data on diagnostic relevance, the concentrations are recommended by the SSC/ISTH Platelet Physiology Subcommittee Working Group (11).

For ADP, the use of 2 μM is recommended, with higher concentrations if no aggregation occurs. However, in patients with delta-storage pool disorders or primary secretion defects, ADP concentrations of 2 to 4 μM may result in abnormal aggregation [16], but aggregation may be normal at higher concentrations. In addition, there is no consensus on whether the absence of aggregation in a 2 μM ADP-induced platelet sample is pathologically significant.

To address this issue, this study aimed to evaluate the effectiveness of different platelet agonists and their concentrations in diagnosing PFDs using LTA. Specifically, we compared LTA results from patients with a history of bleeding tendencies to those from healthy blood donors.

## 2 Methods

This study consists of 2 parts: a retrospective analysis of patient data and a prospective study involving healthy blood donors.

### 2.1 Retrospective study

The retrospective study included patients who attended the Hemostasis Outpatient Department of the University Hospital Leipzig between 2017 and 2019 for diagnostic evaluation of a bleeding tendency. Patients were eligible for inclusion if they underwent LTA as part of their diagnostic work-up. Exclusion criteria were age ≥75 years, pregnancy, use of antithrombotic or antiplatelet medication, use of nonsteroidal anti-inflammatory drugs within 7 days prior to testing, active cancer, and a known bleeding disorder. Patients with known organic diseases (eg, chronic renal failure) and not hemostatic causes (eg, vitamin C deficiency, connective tissue disease, amyloidosis) and evidence of acute-phase reactions, such as infection or systemic inflammation, were ruled out.

In addition to LTA, patients underwent comprehensive stepwise coagulation testing, including assessments of prothrombin time, activated partial thromboplastin time, fibrinogen, thrombin time, von Willebrand factor (VWF) activity, VWF antigen, VWF collagen binding, VWF multimers, factor VIII, and factor XIII. Additional investigations for patients with suspected platelet dysfunction comprised flow cytometry, immunofluorescence microscopy, and genetic testing in select cases [6]. Prior to blood sampling, patients completed the standardized bleeding questionnaire of the University Hospital Leipzig, Germany (Supplementary Table S1) , which is based on the questionnaire published by Koscielny et al. [17] Furthermore, relevant information from patient records, including age, sex, available diagnoses, and comorbidities, were collected.

Patients were divided into 2 groups: those for whom a formal bleeding disorder was established from the results of their diagnostic work-up, and patients for whom no pathological findings could be found in laboratory analysis (BDUC) [1,2].

### 2.2 Prospective study

The prospective study included healthy adults who donated blood at the Institute of Transfusion Medicine, University Hospital Leipzig, Germany between February and May 2020. Donors were excluded if they were <18 years old, were taking antithrombotic or antiplatelet medication, nonsteroidal anti-inflammatory drugs (NSAIDs) or metamizole, had a known bleeding disorder, or a positive bleeding history. To ensure comparability with the retrospective patient group, blood donors also completed the same standardized bleeding questionnaire used in the retrospective study (Supplementary Table S1).

### 2.3 Questionnaire

Diagnostic work-up was performed for patients who scored ≥1 point on the bleeding questionnaire [17]. Subsequently, we retrospectively converted the data from the bleeding questionnaire to the ISTH Bleeding Assessment Tool (BAT) score using the ISTH interpretation aid from the ISTH/SSC [18; see Supplementary Data S1 therein] and medical histories, obtained from doctors’ letters and documented visits.

In accordance with the ISTH guidelines [15], a score of ≥4 was considered “abnormal” for men, and ≥6 was considered “abnormal” for women. Blood donors with ISTH-BAT scores at this threshold of abnormality were categorized as having a positive bleeding history and were excluded from the study [18].

### 2.4 LTA

LTA was performed using the Platelet Aggregation Profiler (PAP-8E, möLab GmbH). Blood was centrifuged at 170 × *g* for 10 minutes to generate platelet-rich plasma (PRP), and a platelet count was determined via an automated cell counter (XN-9000 Analyzer Sysmex). Platelet-poor plasma was then prepared as a blank control by further centrifuging the plasma at 1800 × *g* for 20 minutes after removal of the PRP.

After centrifugation, PRP was left at room temperature for 15 minutes. Baseline tracing was observed for 1 minute before aggregation was induced by the addition of ADP, AA, and EPI to 225 μL of PRP at 37 °C. The aggregation process was monitored for 17 minutes, and both final aggregation (FA) and maximum aggregation (MA) were recorded.

All LTA curves were independently reviewed by 2 hemostaseologists, and MA and FA values were extracted for analysis to enable clear comparison between groups. In-house reference ranges for MA (63%-96%) were established from LTA results of 120 healthy adult volunteers tested at our institution using identical instruments, reagents, and preanalytical conditions; values ≤63% were classified as abnormal based on the 97.5% CI.

### 2.5 Blood sampling and laboratory measurement

We followed the guideline recommendations for blood sampling and took precautions to minimize platelet activation (no or minimal venostasis, 21 gauge needle) using 3.8% citrate tubes (Sarstedt) [14]. Blood samples from patients who were attending the coagulation clinic for diagnostic work-up were analyzed using PAP-8 at the Institute of Laboratory Medicine, Leipzig within 4 hours. For LTA, the following inductors and concentrations were used: ADP (2 μM, 5 μM, and 20 μM), AA (1 mM), EPI (5 mM), Collagen (1.9 mM), Ristocetin (1.2 mM).

For the prospective part of our study, blood samples from healthy individuals who donated blood to the Institute of Transfusion Medicine were drawn according to current standards [19] from the initial 50 mL of the blood donation. Blood samples were analyzed within 4 hours at the hemostasis research laboratory of Leipzig University. Consequently, the procedure for LTA in terms of workflow, processing, and measurement time (all objective parameters) in the research laboratory was adapted to that of the Institute of Laboratory Medicine Leipzig. In the research laboratory, LTA was measured after induction with ADP (2 μM, 5 μM), AA (1 mM, 1.5 mM), and EPI (5 μM, 10 μM). As a cutoff, we used in-house reference values (63% - 96%). According to those, all values ≤63 were considered abnormal.

### 2.6 Statistical analysis

Statistical analysis was conducted using SPSS version 27 (SPSS Inc). The Shapiro Wilk test was used to check for normal distribution. The quantitative variables were analyzed by testing the descriptive statistics in the form of median and IQR. Normally distributed data were expressed as mean values with SD. Additionally, LTA results were calculated for each agonist and its concentrations in quantiles (5, 25, 50, 75, and 95) for each group.

We did not apply Bonferroni correction, as the analyses were predefined, exploratory, and not statistically independent (eg, different concentrations of the same agonist). Given this interdependence, applying a strict Bonferroni adjustment would have disproportionately increased the risk of type II error, thereby masking potentially relevant biological signals.

The analysis was based on both absolute and percentage values. Group comparisons were performed using Pearson’s chi-squared test or Fisher’s exact test. *P* < .05 was considered statistically significant.

### 2.7 Ethical considerations

The study was approved by the University of Leipzig’s Ethics Committee (reference: 384/19-ek) and conducted in accordance with the Declaration of Helsinki. The inclusion of retrospective patients in the study was covered by the ethics committee as a retrospective chart review. The cohort of the prospective study (blood donors) provided informed consent prior to inclusion in the study.

## 3 Results

### 3.1 Patient characteristics

#### 3.1.1 Retrospective cohort

A total of 829 LTA measurements were performed in 649 patients at the Institute of Laboratory Medicine between January 2017 and December 2019. After applying the exclusion criteria, 407 patients were excluded from the study. The remaining 242 patients were divided into 2 groups for analysis: those who were subsequently diagnosed with a bleeding disorder that explained their bleeding tendencies (*n* = 69); and those for whom no diagnosis could be established (*n* = 173, BDUC). Among the individuals who had been diagnosed with a bleeding disorder, the most common diagnoses were von Willebrand disease (*n* = 22), acquired or hereditary factor deficiency (*n* = 20), PFD (*n* = 20), fibrinogen deficiency (*n* = 5), and other organic diseases (eg chronic renal failure) leading to bleeding tendency (*n* = 2).

The group of patients with BDUC comprised of 173 patients who were not diagnosed with a bleeding disorder despite presenting with clinical symptoms and undergoing comprehensive testing for a bleeding disorder. The median age of this group was 41 years (IQR, 33-53; range 49). The cohort comprised 29 males (16.8%) and 144 females (83.2%).

#### 3.1.2 Prospective cohort

Blood samples were collected from 176 healthy blood donors (median age 44 years [IQR, 33-56; range 48], 53 females [38.7%]). Of those, 137 were included in the study. Of the 39 donors excluded from the study, 2 had taken an NSAID before blood donation, 1 had a positive bleeding questionnaire, another subject had a known bleeding disorder (not relevant for blood donation), and 35 donors’ samples returned inadequate LTA measurements. Most of these measurements failed due to preanalytic problems (eg, lipid plasma, analysis >4 hours after collection).

The Figure shows a flow chart that summarizes the number of patients/donors included in the study and reasons for exclusion.

**Figure:**
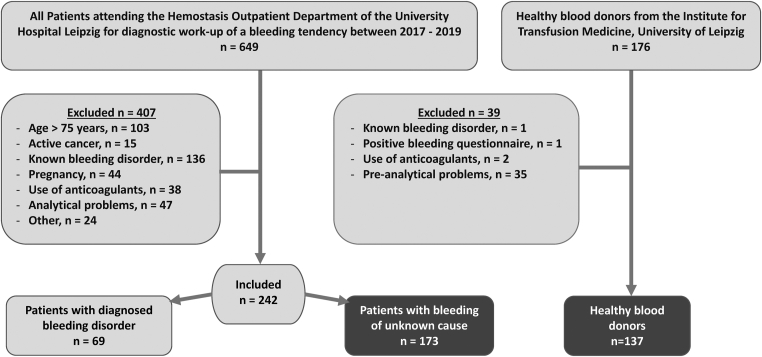
Flow chart of patients included in the study.

### 3.2 Comparison of patients with BDUC and healthy blood donors

Following the administration of 1 mM AA, the proportion of abnormal platelet aggregation in patients with BDUC was significantly higher than that in healthy blood donors (FA: 19.1% vs 4.4%, *P* < .001). After induction with 5 μM EPI, 9.2% of the patients with BDUC and 5.8% of the blood donors had a pathologic FA in LTA result (*P* = .27). The stimulation with 2 μM ADP resulted in an abnormal FA in 49.6% of the blood donors and 29.5% of the patients with BDUC (*P* < .001). Furthermore, a higher proportion of blood donors also exhibited an abnormal MA (43.8% vs 24.3%, *P* < .001) in response to 2 μM ADP. When using 5 μM ADP, the proportion of healthy blood donors with abnormal platelet function was significantly higher than that of patients with BDUC (22.6% vs 12.1%, *P* = .01). The results are summarized in Table 1.

**Table 1:** Proportion of abnormal LTA results (*n*, %) with different agonists in healthy blood donors and patients with bleeding disorder of unknown cause, only the first measurement was included.

Inductor	Endpoint	Healthy blood donor (*n* = 137)	Patients with BDUC (*n* = 173)	*P*
AA, 1 mM	MA	5 (3.6)	32 (18.5)	< .001
	FA	6 (4.4)	33 (19.1)	< .001
AA, 1.5 mM	MA	3 (2.1)	n.a.	
	FA	3 (2.1)	n.a.	
EPI, 5 μM	MA	7 (5.1)	14 (8.1)	.30
	FA	8 (5.8)	16 (9.2)	.27
ADP, 2 μM	MA	60 (43.8)	42 (24.3)	< .001
	FA	68 (49.6)	51 (29.5)	< .001
ADP, 5 μM	MA	8 (5.8)	8 (4.6)	.63
	FA	31 (22.6)	21 (12.1)	.01
ADP, 20 μM	MA	2 (1.5)	4 (2.3)	.70
	FA	18 (13.1)	13 (7.5)	.10

AA, arachidonic acid; ADP, adenosine diphosphate; BDUC, bleeding disorder of unknown cause; EPI, epinephrine; FA, final aggregation; LTA, light transmission aggregometry; MA, maximum aggregation; n.a.: not assessed.

### 3.3 Case matches

To identify differences by age, subjects in both cohorts were divided into the following groups: 18 to 29 years, 30 to 39 years, 40 to 49 years, 50 to 59 years, and 60 to 69 years.

In the 30-39 year age group, the proportion of patients with BDUC with abnormal FA after induction with 1 mM of AA was significantly higher than that observed in blood donors (23.1% vs 3.6%, *P* = .03). In the 40-49 year age group, 13.5% of patients with BDUC and 52.9% of blood donors showed an abnormal FA after induction with 2 μM ADP (*P* < .001), while 2.7% of patients and 20.6% of blood donors had abnormal MA after induction with 5 μM ADP (*P* = 0.002). No significant differences were observed for the other age groups, agonists, or concentrations (Supplementary Table S2).

### 3.4 Patients with multiple LTA measurements

Multiple LTA measurements were performed in 41 patients with BDUC. There were 3 reasons why some patients underwent multiple tests: abnormalities in the LTA results, analysis errors, and ingestion of anticoagulants or dietary supplements. Despite a pronounced bleeding tendency, the LTA results of those patients repeatedly revealed inconclusive findings. In cases where a definitive diagnosis was not reached at the time of data collection, up to 7 follow-up measurements were taken over an extended period in the outpatient clinic.

After induction with 1 mM of AA, 6 patients with BDUC exhibited initial LTA values that were below the reference range, but all follow-up measurements were within the normal range. In 4 BDUC patients, initial values were within the reference range, but follow-up measurements were outside the reference range.

After induction with 5 μM EPI, 5 patients with BDUC had abnormal initial measurements, but the results of the follow-up tests were within the normal range.

In 12 patients with BDUC, aggregation with 2 μM ADP was outside the reference range at the initial measurement. Of those, 2 patients had abnormal values in all follow-up measurements, 7 patients showed normal and abnormal values, and 3 patients had only normal follow-up measurement results.

Aggregation was outside the reference range during the initial measurement in 4 patients with BDUC after induction with 5 μM ADP, but only 1 patient had abnormal values in later follow-up measurements. Three patients with BDUC had abnormalities in the first measurement with 20 μM of ADP but were within the reference range when tested again.

### 3.5 ISTH-BAT score to predict LTA

Analysis of ISTH-BAT scores in 173 patients with BDUC yielded an overall median score of 4. When analyzed by sex, 29 male patients showed a median of 3 (IQR, 2-4), while 144 female patients had a median of 4 (IQR, 3-5).

Although presenting with a normal ISTH-BAT score, 30.1% of BDUC patients had abnormal FA in LTA results with 2 μM ADP. In contrast, only 2.5% of patients showed an abnormal FA with 20 μM ADP, despite having an abnormal ISTH-BAT score. The data are summarized in Table 2.

**Table 2:** ISTH-BAT score and results of final aggregation in LTA according to activator, *n* (%).

Inductor	ISTH-BAT normal		ISTH-BAT abnormal		*P*
	LTA		LTA		
	Normal	Abnormal	Normal	Abnormal	
AA, 1 mM	104 (78.2)	29 (21.8)	36 (90.0)	4 (10.0)	.10
EPI, 5 μM	119 (89.5)	14 (10.5)	38 (95.0)	2 (5.0)	.29
ADP, 2 μM	93 (69.9)	40 (30.1)	29 (72.5)	11 (27.5)	.75
ADP, 5 μM	115 (86.5)	18 (13.5)	37 (92.5)	3 (7.5)	.31
ADP, 20 μM	121 (91.0)	12 (9.0)	39 (97.5)	1 (2.5)	.17

AA, arachidonic acid; ADP, adenosine diphosphate; EPI, epinephrine; ISTH-BAT, International Society on Thrombosis and Haemostasis Bleeding Assessment Tool; LTA, light transmission aggregometry.

The sensitivity, specificity, negative predictive value (NPV) and positive predictive value (PPV) of an abnormal ISTH-BAT score to predict abnormal LTA were calculated using the data in Table 2. The specificity and NPV of an abnormal ISTH-BAT in predicting an abnormal LTA were >74%, while the sensitivity and PPV were <30%. The highest specificity and NPV were observed with EPI and ADP at concentrations of 5 μM and 20 μM. All findings are presented in Table 3.

**Table 3:** Sensitivity, specificity, NPV, and PPV of an abnormal ISTH-BAT to predict abnormal LTA.

Inductor	Sensitivity (%)	Specificity (%)	NPV (%)	PPV (%)
AA, 1 mM	12.1	74.3	78.2	10
EPI, 5 μM	12.5	75.8	89.5	5.0
ADP, 2 μM	21.6	76.2	70.0	27.5
ADP, 5 μM	14.3	75.7	86.5	7.5
ADP 20 μM	7.7	75.6	91	2.5

AA, arachidonic acid; ADP, adenosine diphosphate; EPI, epinephrine; ISTH-BAT, International Society on Thrombosis and Haemostasis Bleeding Assessment Tool; LTA, light transmission aggregometry; NPV, negative predictive value; PPV, positive predictive value.

## 4 Discussion

The diagnostic significance of recommended agonists and their concentrations remains unclear, as abnormal aggregation may normalize at higher concentrations. The aim of this study was to evaluate the effectiveness of different platelet agonists and concentrations by comparing LTA results from patients with BDUC and healthy blood donors. This study reveals a high proportion of abnormal LTA results in patients with BDUC and healthy blood donors after induction with low concentrations of ADP and AA, results that question the recommendation of low agonist concentrations.

In accordance with the prevailing international guidelines, LTA remains the gold standard for the diagnosis of PFDs [5]. The ISTH guideline specifies a standard set of agonists and their corresponding concentrations to be utilized in conjunction with LTA. ADP, a natural agonist of platelet aggregation, inhibits the cAMP signaling pathway of cells through P2Y1 and P2Y12 receptors. Abnormal aggregation under ADP may indicate diseases such as P2Y12 receptor defects or storage pool disease [10]. EPI promotes aggregation via α2-adrenergic receptors, thereby enabling the detection of storage pool disease and other diseases [10]. AA primarily enhances platelet adhesion by activating thromboxane A2 and is irreversibly inhibited by acetylsalicylic acid [10].

Pathological aggregation in response to 1 mM AA was observed in 4% of healthy blood donors, compared to 19% of patients with BDUC. Increasing AA concentrations to 1.5 mM reduced the rate of pathological findings in blood donors to 2.1%. This suggests that higher AA concentrations (>1.5 mM) may reduce the rate of false-positive results and improve the specificity of LTA in detecting clinically relevant defects in the AA signaling pathway. Unfortunately, LTA results using 1.5 mM AA were unavailable for the BDUC cohort. The increased proportion of abnormal findings at 1 mM AA among patients with BDUC may reflect an aspirin-like defect. However, this finding could not be reproduced in most patients, suggesting that the concentration of 1.0 mM AA may be too low to draw a definitive conclusion about a relevant PFDs. However, some patients and healthy volunteers may not have declared taking NSAIDs, which could have led to false-positive results.

For 2 μM ADP, we found pathological aggregation in >20% of the BDUC patients and in even more healthy blood donors. This raises questions about the diagnostic significance of this agonist concentration, which is currently recommended in the ISTH guideline as the first concentration to be used. Based on the ISTH recommendation, Cattaneo et al. [9] recommend 2 μM ADP but state that higher concentrations of ADP should be used if abnormal results with 2 μM are obtained. Harrison [4] reported the British Society of Hematology recommendation, starting with 2.5 μM ADP. The proportion of abnormal aggregation decreased with 5 μM ADP to about 5% for MA in both cohorts and to 22.6% and 12.1% for FA in healthy blood donors and BDUC patients, respectively. This indicates that 5 μM ADP should be sufficient to rule out relevant platelet function defects, and MA should be used rather than FA.

Although elevating the ADP concentration to 20 μM further reduced the rate of pathological aggregation in terms of MA and FA, this higher agonist dose may decrease sensitivity for detecting mild PFDs, since MA >70% when using 10 μM ADP is an established threshold for identifying low responders to clopidogrel [20].

In contrast, with 5 μM EPI, only 5.8% of the patients with BDUC and 9.2% of the healthy blood donors showed abnormal aggregation in our study. The proportion of abnormal results with low-dose EPI was higher in the study of Alessi et al. [12], which is why they proposed 25 μM EPI to distinguish poor responders from healthy volunteers.

This highlights that the minimum dose to produce MA in platelets remains unknown. The analysis of patients with BDUC with multiple measurements further supports this fact. Even in cases in which substantial fluctuations were observed, these could be attributed more to intraindividual factors and laboratory variations rather than to pathologic platelet dysfunction.

A recent multicenter evaluation of LTA reagents revealed—in agreement with our results—a greater variability at low agonist concentrations as well as a significant influence of source of used activators [21]. In addition, the GTH-based THROMKID-Plus Study Group performed an interlaboratory trial and reported high internal variability of the laboratory induction agents in terms of reagent type, concentrations, and pathological cutoff values [22]. During the effort to harmonize LTA in the Netherlands, data revealed that standardization was not effective for low ADP concentrations, probably due to higher batch dependence [23]. This underscores the importance of questioning the diagnostic value of 2 μM ADP.

Our data suggest that 2 μM could be insufficient to induce a stable platelet response and indicate that MA with 5 μM ADP may be the optimal concentration. Therefore, the recommendation of the ISTH to use 2 μM ADP for the first investigation in patients with suspected PFD and after abnormal results, they should be re-evaluated with higher doses [14].

Although our retrospective cohort also included a small group of patients with confirmed PFD, the heterogeneity of underlying defects (eg, storage pool disease, receptor defects) and the limited sample size precluded a meaningful statistical comparison with BDUC patients. Such a comparison would be valuable in future prospective studies to determine whether pathological findings at low agonist concentrations have clinical relevance across different patient categories.

Otahbachi et al. [24] demonstrated that the process of aggregation is influenced by a number of intraindividual factors including age and sex. Our attempt to divide both cohorts into age groups for the analysis did not allow a comparison by sex due to the unequal distribution in both cohorts. It is noteworthy that among patients with BDUC aged 30 to 39, abnormal platelet function with AA was more frequent than in patients with BDUC of any other age group. Conversely, in the 40 to 49 age range, patients with BDUC showed a significantly reduced incidence of abnormal aggregation when 2 μM of ADP was utilized as the agonist. In their investigation of the influence of age on aggregation, Vilén et al. [25] included a group of healthy adults with a similar sex distribution. A significant correlation was observed between primary aggregation (area under the curve) and age, in both men and women, particularly when stimulated at concentrations of 0.2 to 1.0 μM ADP. The present study revealed that a concentration of 2 μM ADP (double the concentration used in Vilén’s study [25]) resulted in abnormal aggregation rates ranging from 46.2% to 60%. Interestingly, the 50 to 59 age group showed a lower incidence of abnormal aggregation, at 33.3%, than other age groups. The available evidence indicates that age may be a contributing factor in platelet aggregation. However further research is necessary to substantiate this hypothesis.

In addition to LTA and other laboratory tests, questionnaires can be used to assess the need for further investigation of a bleeding tendency [8]. The ISTH-BAT is recognized as a validated clinical instrument, particularly for the diagnosis of von Willebrand disease and PFD [26]. Lowe et al. [27] concluded in 2023, that although the ISTH-BAT score was not predictive for PFD, the questionnaire proved to be a useful tool for documenting bleeding history. In contrast, Gresele et al. [3] documented lower MA in LTA in patients with known PFD and a positive bleedings score. However, these results were mainly driven by patients with Glanzmann thrombasthenia and CalDAG-GEFI deficiency and remained significant only for TRAP after exclusion of those patients. Further, Ambaglio et al. [28] reported in 1503 preoperative patients that the ISTH-BAT and laboratory screening tests did not accurately detect mild bleeding disorders in select patients. Our study showed very low sensitivity and PPV of an abnormal ISTH-BAT score to predict abnormal LTA; only NPV was acceptable for EPI and the higher concentration of ADP, which supports this hypothesis.

### 4.1 Limitations

The retrospective nature of the patient data may have led to potential biases, such as incomplete records or unmeasured confounders, which could have influenced the results of the study. The relatively small sample size, most notably in the prospective cohort of healthy blood donors, limits the generalizability of the findings, particularly for the comparison between age groups. Although most patients were Caucasian, we did not collect data on the ethnicity of all patients and blood donors, which may have further influenced the results. Since the blood donor group did not undergo a control measurement, it was assumed in this study that the information provided by the blood donors and their LTA results represent their platelet function in a stable and consistent manner. In this context, it should be acknowledged that studies have shown that platelet function is sensitive to inter- and intraindividual daily variations [24], as we have seen in our patients with BDUC with multiple measurements. It should be noted that the retrospective conversion from the Koscielny questionnaire to the ISTH-BAT score may result in apparently normal ISTH-BAT values, as some items are weighted differently or are not directly transferrable between the 2 instruments. Due to the heterogeneity of underlying defects and the limited sample size, a direct statistical comparison between patients with BDUC and PFDs were considered underpowered and potentially misleading. Some patients with no significant clinical history and normal initial test results did not receive repeated testing, which might have resulted in the missed diagnosis of a mild bleeding disorder. For the simplification of the comparison between study groups, only MA and FA values of LTA were reported. Finally, multiple statistical comparisons were performed without Bonferroni correction. This approach was chosen due to the exploratory and interdependent nature of the analyses, but it may have increased the risk of false-positive findings; accordingly, our results should be interpreted with caution.

### 4.2 Conclusion

LTA remains a widely used and accepted method for detecting PFDs. These findings suggest that low agonist concentrations, as currently recommended in LTA protocols, may have limited specificity and sensitivity in identifying some platelet function abnormalities in patients with BDUC. Further studies integrating standardized LTA with genetic testing are warranted to clarify whether adjusted protocols could enhance diagnostic yield without compromising specificity.
