# Supplementary material for: Comparison of light transmission aggregometry in patients with bleeding disorder of unknown cause and healthy blood donors
Source: Res Pract Thromb Haemost. 2025 Oct 10;9(7):103214. doi: 10.1016/j.rpth.2025.103214 (PMC12621560; doi:10.1016/j.rpth.2025.103214)
Supplement: Supplementary Tables [file mmc1.pdf]

Supplementary material

Supplementary Table S1: Bleeding questionnaire according to Koscielny at al. used in the study

Bleeding disorder?	No	Yes	Which one?
Epistaxis?	No	Yes	How often? _____<br>Which treatment helped?___________<br>Was it necessary to transfuse blood?
Do you have a tendency to bruise? Disposition for petechiae?	No	Yes	Spontaneous or traumatic?<br>Where?<br>Arms/Legs: _______<br>Upper body: ________
Bleeding of a joint, muscle or soft tissue?	No	Yes	Spontaneous or traumatic ________<br>Where? ________
Prolonged bleeding after cuts or abrasion?	No	Yes	Where? _______<br>Which device of hemostasis was needed?
Did you have gum bleeding or prolonged bleeding after dental extraction?	No	Yes	date: ____________<br>How often? ____________<br>Which device of hemostasis was needed?
Bleeding complication after surgery or birth?	No	Yes	What?_______________________<br>date: ___________________<br>How was the bleeding treated?
Did you discover a wound healing deficit?	No	Yes	For which injuries?
Does a family member have a bleeding tendency?	No	Yes	Who? ____________<br>Which one? ____________
Intake of acetylsalicylic acid, ibuprofen, ginger or other anticoagulation medication?	No	Yes	Which one? ______________<br>How often? ____________________<br>When was the last time?_______________
Do you have prolonged or heavier menstruation?	No	Yes	Duration:<br>Change of sanitary pad/tampons every ____ hours

Supplementary Table S2: Proportion of patients with abnormal LTA values in different age groups

			BDUC		Blood donors		
**Age**			n	%	n	%	p
18 - 29	**AA 1mM**	MA	6 / 26	23.1%	1 / 26	3.8%	0.099
		FA	6 / 26	23.1%	2 / 26	7.7%	0.248
	**EPI 5µM**	MA	3 / 26	11.5%	2 / 26	7.7%	1.000
		FA	4 / 26	15.4%	2 / 26	7.7%	0.668
	**ADP 2µM**	MA	6 / 26	23.1%	12 / 26	46.2%	0.080
		FA	7 / 26	26.9%	12 / 26	46.2%	0.150
	**ADP 5µM**	MA	1 / 26	3.8%	0 / 26	0.0%	1.000
		FA*	3 / 26	11.5%	8 / 26	30.8%	0.090
	**ADP 20µM**	MA	0 / 26	0.0%	0 / 26	0.0%	-
		FA	3 / 26	11.5%	5 / 26	19.2%	0.703
30-39	**AA 1mM**	MA*	11 / 52	21.2%	1 / 28	3.6%	0.048
		FA*	12 / 52	23.1%	1 / 28	3.6%	0.027
	**EPI 5µM**	MA	5 / 52	9.6%	1 / 28	3.6%	0.659
		FA	5 / 52	9.6%	2 / 28	7.1%	1.000
	**ADP 2µM**	MA	16 / 52	30.8%	13 / 28	46.4%	0.165
		FA	21 / 52	40.4%	15 / 28	53.6%	0.258
	**ADP 5µM**	MA	3 / 52	5.8%	4 / 28	14.3%	0.232
		FA	7 / 52	13.5%	5 / 28	17.9%	0.744
	**ADP 20µM**	MA	1 / 52	1.9%	0 / 28	0.0%	1.000
		FA	4 / 52	7.7%	5 / 28	17.9%	0.265
40-49	**AA 1mM**	MA	3 / 37	8.1%	0 / 34	0.0%	0.241
		FA	3 / 37	8.1%	0 / 34	0.0%	0.241
	**EPI 5µM**	MA	1 / 37	2.7%	0 / 33	0.0%	1.000
		FA	1 / 37	2.7%	0 / 33	0.0%	1.000
	**ADP 2µM**	MA*	3 / 37	8.1%	17 / 34	50%	< 0.001
		FA*	5 / 37	13.5%	18 / 34	52.9%	< 0.001
	**ADP 5µM**	MA	1 / 37	2.7%	2 / 34	5.9%	0.604
		FA*	1 / 37	2.7%	7 / 34	20.6%	0.024
	**ADP 20µM**	MA	0 / 37	0.0%	1 / 34	2.9%	0.479
		FA	1 / 37	2.7%	3 / 34	8.8%	0.344
50 - 59	**AA 1mM**	MA	7 / 39	17.9%	1 / 24	4.2%	0.141
		FA	7 / 39	17.9%	1 / 24	4.2%	0.141
	**EPI 5µM**	MA	3 / 39	7.7%	1 / 24	4.2%	1.000
		FA	3 / 39	7.7%	1 / 24	4.2%	1.000
	**ADP 2µM**	MA	8 / 39	20.5%	6 / 24	25%	0.677
		FA	9 / 39	23.1%	8 / 24	33.3%	0.373
	**ADP 5µM**	MA	3 / 39	7.7%	0 / 24	4%	0.281
		FA	8 / 39	20.5%	3 / 24	12.5%	0.509
	**ADP 20µM**	MA	3 / 39	7.7%	0 / 25	0.0%	0.281
		FA	4 / 39	10.3%	3 / 24	12.5%	1.000
60-69	**AA 1mM**	MA	5 / 19	26.3%	2 / 25	8%	0.210
		FA	5 / 19	26.3%	2 / 25	8%	0.210
	**EPI 5µM**	MA	2 / 19	10.5%	3 / 25	12%	1.000

	FA	3 / 19	15.8%	3 / 25	12%	1.000
**ADP 2µM**	MA	9 / 19	47.4%	12 / 25	48%	0.967
	FA	9 / 19	47.4%	15 / 25	60%	0.405
**ADP 5µM**	MA	0 / 19	0.0%	2 / 25	8%	0.498
	FA	2 / 19	10.5%	8 / 25	32%	0.148
**ADP 20µM**	MA	0 / 19	0.0%	1 / 25	4%	1.000
	FA	1 / 19	5.3%	2 / 25	8%	1.000
